# Serum Proteomic and Oxidative Modification Profiling in Mice Exposed to Total Body X-Irradiation

**DOI:** 10.3390/antiox11091710

**Published:** 2022-08-30

**Authors:** Masaru Yamaguchi, Yota Tatara, Eka Djatnika Nugraha, Yoshiaki Sato, Tomisato Miura, Masahiro Hosoda, Mukh Syaifudin, Shinji Tokonami, Ikuo Kashiwakura

**Affiliations:** 1 Hirosaki University Graduate School of Health Sciences, 66-1 Hon-cho, Hirosaki 036-8564, Aomori, Japan; 2 Hirosaki University Graduate School of Medicine, 5 Zaifu-cho, Hirosaki 036-8562, Aomori, Japan; 3 The Research Center for Safety, Metrology, and Nuclear Quality Technology (PRTKMMN), Research Organization for Nuclear Energy, National Research and Innovation Agency of Indonesia (BRIN), JI. Lebak Bulus Raya No. 49, Jakarta Selatan 12440, DKI Jakarta, Indonesia; 4 Institute of Radiation Emergency Medicine, Hirosaki University, 66-1 Hon-cho, Hirosaki, Aomori 036-8564, Japan; 5 Research Center for Radioisotope, Radiopharmaceutical and Biodosimetry Technology, Research Organization for Nuclear Energy, National Research and Innovation Agency, Kw. Puspiptek, Setu, Tangerang Selatan 15312, Banten, Indonesia

**Keywords:** total body irradiation, proteomic analysis, oxidative modification profiling, serum albumin, amino acid sequences, radiation biomarker

## Abstract

The details of the dose-dependent response of serum proteins exposed to ionizing radiation, especially the oxidative modification response in amino acid sequences of albumin, the most abundant protein, are unknown. Thus, a proteomic analysis of the serum components from mice exposed to total body X-irradiation (TBI) ranging from 0.5 Gy to 3.0 Gy was conducted using LC-MS/MS. The analysis of oxidative modification sequences of albumin (mOMSA) in TBI mouse serum revealed significant moderate or strong correlations between the X-irradiation exposure dose and modification of 11 mOMSAs (especially the 97th, 267th and 499th lysine residues, 159th methionine residue and 287th tyrosine residues). In the case of X-irradiation of serum alone, significant correlations were also found in the 14 mOMSAs. In addition, a dose-dependent variation in six proteins (Angiotensinogen, Odorant-binding protein 1a, Serine protease inhibitor A3K, Serum paraoxonase/arylesterase 1, Prothrombin and Epidermal growth factor receptor) was detected in the serum of mice exposed to TBI. These findings suggest the possibility that the protein variation and serum albumin oxidative modification responses found in exposed individuals are important indicators for considering the effects of radiation on living organisms, along with DNA damage, and suggests their possible application as biomarkers of radiation dose estimation.

## 1. Introduction

Very recently we reported on a proteomic analysis and oxidative modification profiling of serum collected from residents of a newly discovered high-level natural background radiation area (HBRA, annual effective dose of approximately 50 mSv y^−1^) and normal-level background radiation area (NBRA, 1.22 mSv y^−1^) in Mamuju, Indonesia [1]. A proteomic analysis showed that the apolipoprotein B-100 and hemoglobin subunit α1 expression of residents in the HBRA was significantly lower than that of residents in the NBRA. In addition, a total of 270 oxidation-mediated modification sites were identified in the amino acid sequence of human serum albumin (HSA) by liquid chromatography-tandem mass spectrometry (LC-MS/MS). Among these, four specific amino acid sequences of HSA showed a dose-dependent oxidative modifications. Notably, the 162nd and 356th tyrosine residues and 111th and 470th methionine residues were found. None of these findings have been reported in humans exposed to chronic low-dose radiation. This can be used as a biomarker not only for the assessment of the presence or absence of radiation exposure but also for dose prediction of chronic radiation exposure in living organisms. These results suggest that traces of radiation exposure are recorded in serum albumin and that there is a possibility of a new methodology that can evaluate biological responses below 100 mSv.

Regarding the health effects of chronic low-dose radiation exposure, epidemiological studies of human populations, such as occupational studies of nuclear workers, are not as clear regarding whether low-dose-rate exposure results in lower risks than seen among Japanese atom bomb survivors who were acutely exposed to radiation [2]. In addition, the UNSCEAR report showed that epidemiological studies in several regions of the world (Ramsar, Yangjiang, Kerala and Guarapari) reported no correlation between radiation exposure and cancer rate or mortality in areas with high natural background radiation [3], indicating that the effect of low dose rates on health and the cancer risk after exposure to ionizing radiation is still unclear. Tang et al. also reported that the mechanisms of low-dose ionizing radiation (≤100 mSv) or low-dose-rate ionizing radiation (<6 mSv/h)—induced health effects are poorly understood [4]. Issues related to the health effects of low doses require further research in the future.

The annual effective dose shown in the previous report was estimated as the accumulation of the dose from external exposure (environmental gamma radiation) and internal exposure (mainly through breathing of indoor radon) based on our previous reports. This radioactivity was mainly derived from uranium (^238^U), thorium (^232^Th), radon (^222^Rn), thoron (^220^Rn) and their progeny contained in soil [5,6]. However, do the proteomic changes observed in residents living under chronic long-term low-dose radiation exposure also occur with a single acute high-dose radiation exposure, such as a radiation exposure accident? Furthermore, the details of whether there is a dose-dependent response are unknown. In particular, prodromal symptoms seen in patients within 1 to 2 days after acute radiation exposure of ≥1 Gy may include symptoms such as loss of appetite, nausea, vomiting (>2 Gy), and diarrhea, making it easy to confirm the biological response to radiation exposure [7]. As pointed out by Shin et al., the effects of low-dose radiation, which many experimental studies consider to be defined as <0.5 Gy, are subtle, and the absence of reliable biological markers has been an obstacle [8,9]. With the rapid progress of analytical techniques in recent years, an increasing number of studies have reported on the search of the proteome of exposed individuals [9,10,11,12], and it is expected to be utilized as a biomarker for dose estimation in triage in the event of nuclear or radiological disasters [13,14,15]. However, the details of the relationship between the radiation dose and oxidative modification of serum albumin are unknown. Furthermore, considering its application to radiation accidents and nuclear disasters, it is necessary to verify it in animal experimental models, as it cannot be verified in humans.

In the present study, we analyzed the proteome and oxidative modification profile by LC-MS/MS using mouse serum after 24 h of single total body X-irradiation (0.5–3 Gy), assuming a nuclear disaster/radiation accident.

## 2. Materials and Methods

### 2.1. Animal Experiments

Seven-week-old female C57BL/6JJcl mice were delivered from the breeding facilities of CLEA Japan (Tokyo, Japan). All mice were housed in a conventional clean room at an ambient temperature of 23 °C with 50% relative humidity, and a 12-h light/dark cycle. The mice had ad libitum access to sterilized standard laboratory mouse chow (CLEA Rodent Diet CE-2, CLEA, Tokyo, Japan) and drinking water. After obtaining approval from the animal experiment committee (approval number: G17001), all experiments were conducted according to the legal regulations in Japan and the Guidelines for Animal Experiments, and all efforts were made to minimize the number of animals used and their suffering in this study. After a week of acclimatization, 8-week-old mice were randomly divided into 4 groups with more than 8 mice per group and subjected to varying TBI doses of 0, 0.5, 1 or 3 Gy from X-rays (150 kVp, 20 mA, 0.5-mm aluminum and 0.3-mm copper filters) at a dose rate of 1.0 Gy/min using an MBR-1520R X-ray generator (Hitachi Medical, Tokyo, Japan) with a distance of 450 mm between the focus and the target. The air kerma was monitored with a thimble ionization chamber, which integrated the radiation dose and blocked X-rays when it reached a present dose value. Peripheral blood was harvested by capillary tube 24 h after TBI from the orbital venous plexus of mice after they were anesthetized using isoflurane (Powerful Isoful; Zoetis, London, UK). Samples were placed at room temperature for at least 30 min to allow blood clotting. Serum was collected by centrifugation at 1200× *g* for 10 min and stored at −80 °C until use. In addition, serum collected from non-irradiated mice was subjected to varying TBI doses of 0, 0.5, 1 or 3 Gy from X-rays; incubated at 37 °C for 24 h; and stored at −80 °C until use.

### 2.2. Liquid Chromatography-Tandem Mass Spectrometry (LC-MS/MS) and High-Resolution Multiple Reaction Monitoring (MRM-HR)

The measurement was performed according to a previous report [1]. Briefly, serum proteins were precipitated with acetone and the precipitates were dissolved and denatured with 50% trifluoroethanol. The proteins were reduced and alkylated before trypsinization. Tryptic peptides were analyzed using a TripleTOF6600 mass spectrometer (AB Sciex). A non-labeled quantitative method (SWATH) was used for a serum proteome analysis. The peak areas of peptides were normalized to the sum of the total peak area sum of all measured peptides. The principal component analysis (PCA) and orthogonal partial least square-discriminant analysis (OPLS-DA) were performed using the Simca software program (Infocom Corp, Tokyo, Japan).

The high-resolution multiple reaction monitoring (MRM-HR) method was used to profile oxidative modification of serum albumin. On the basis of the information-dependent acquisition results (data not shown), an assay for MRM-HR experiments was developed using the Skyline software program (MacCoss Lab, University of Washington, Seattle, Washington, DC, USA). The transitions of MRM-HR are shown in Supplementary Table S1. All peak pickings were manually checked after automated matching. The peak areas obtained were normalized by calculating the relative abundance of each modified peptide using the corresponding non-modified peptide.

### 2.3. Statistical Analysis

We used the Origin Pro 2020b software program (Northampton, MA, USA) for Windows to perform the linear and polynomial correlation analysis. Furthermore, the data were analyzed with a one-way ANOVA and Tukey-Kramer or Bonferroni/Dunn multiple comparison tests. Statistical significance in the analysis was all tested using a two-sided *p* value of 0.05. The oxidation modification patterns of amino acids were drawn using BKChem, a freely available chemical drawing program.

## 3. Results

### 3.1. Multivariate Analysis of Serum Proteome of Mice with Different Irradiation Doses

**Figure 1:**
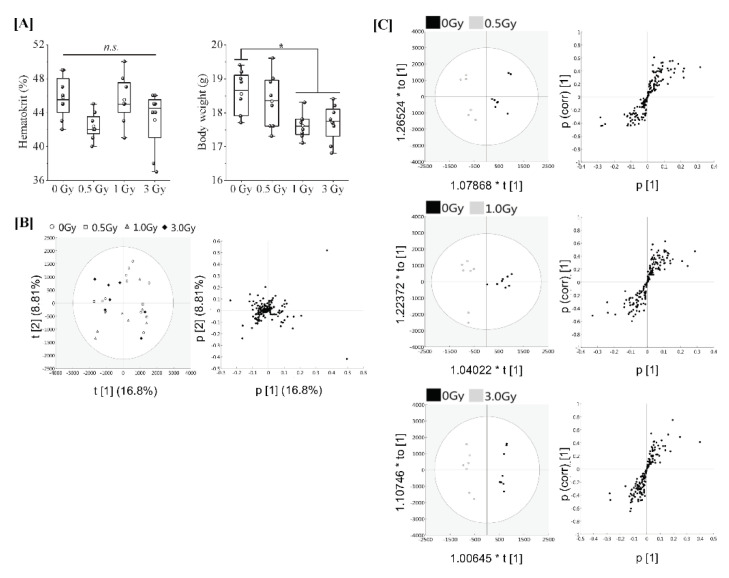
**Proteomic analysis of serum from mice exposed to single TBI in in vivo model.** [**A**] Eight-week-old female C57BL/6JJcl mice were randomly divided into 4 groups with more than 8 mice per group and subjected to varying TBI doses of 0, 0.5, 1 or 3 Gy of X-rays at a dose rate of 1.0 Gy/min. Peripheral blood was harvested 24 h after TBI from the orbital venous plexus of mice and placed at room temperature for at least 30 min to allow blood clotting for serum collection (in vivo model). Body weight changes and haematocrit values at the time of serum collection. Statistically significant differences were evaluated by a one-way ANOVA and the multiple comparison tests; *p* < 0.05 (*). [**B**] PCA score scatter plot of the serum proteome. Each dose treatment group of the samples are represented as shown in the (**left**) figure, respectively. Uncharacterized samples are plotted at the center, and those with features are plotted at a distance from the center (right) figure. Similar features are plotted at close positions. [**C**] The OPLS-DA model to discriminate the serum proteome of each irradiated mouse. The score scatter plot and S-plot are represented. The ellipse in the score scatter plot indicates the Hotelling T2 (0.95) range for each model.

Eight-week-old female C57BL/6JJcl mice were subjected to varying TBI doses of 0.5, 1 or 3 Gy from X-rays, and peripheral blood was harvested 24 h after TBI for serum collection (in vivo model). Regarding animal conditions after 24 h of TBI, as shown in Figure 1A, the body weights of TBI (1 Gy and 3 Gy) mice were significantly decreased in comparison to those of the non-irradiated mice. However, haematocrit values, which indicate the ratio of the total volume of red blood cells to the total blood, did not differ to a statistically significant extent among all groups. To elucidate the effects of each irradiation exposure, LC-MS/MS was used to examine the expression of proteins in the serum in each treatment. Finally, 161 types of protein were identified. The full dataset from all serum samples was subjected to PCA to obtain an overview of the data. The first and second principal component scores were 16.8% and 8.81%, respectively, as shown in Figure 1B (the ellipse represents a 95% tolerance region for the scores based on Hotelling’s T2). There was no evidence of separation among the four classes along the first and second principal components. There were no major outliers. The score scatter plots of the OPLS-DA model in Figure 1C demonstrated satisfactory separation between non-irradiated mice and mice exposed to TBI (0.5 Gy, 1 Gy, or 3 Gy) using one predictive component and one orthogonal component. The above groups were completely separated along the first predictive component. These results indicate that the serum proteome profile can be used to distinguish mice with TBI doses of 0.5 Gy, 1 Gy or 3 Gy from X-rays from non-irradiated mice.

The resultant S-plots using the OPLS-DA model revealed a significant increase in serine protease inhibitor A3K (Serpin A3K) in TBI (1Gy and 3Gy) mice (Table 1) and a further weak dose-dependent correlation was observed when the proteins expressed in non-TBI and TBI mice were compared (Supplemental Figure S1). Angiotensinogen (Serpin A8) and Odorant-binding protein 1a (Odorant-binding protein 1A) were decreased in TBI (0.5 Gy) mice and TBI (1 Gy) mice, respectively. Further serum paraoxonase/arylesterase 1 (PON1), prothrombin, and epidermal growth factor receptor (EGFR) were identified by TBI (3Gy) in addition to Serpin A3K (Table 1). At this time, only PON1 was decreasing.

### 3.2. Oxidative Modification of Serum Albumin (OMSA) under Acute Single Radiation Exposure

**Figure 2:**
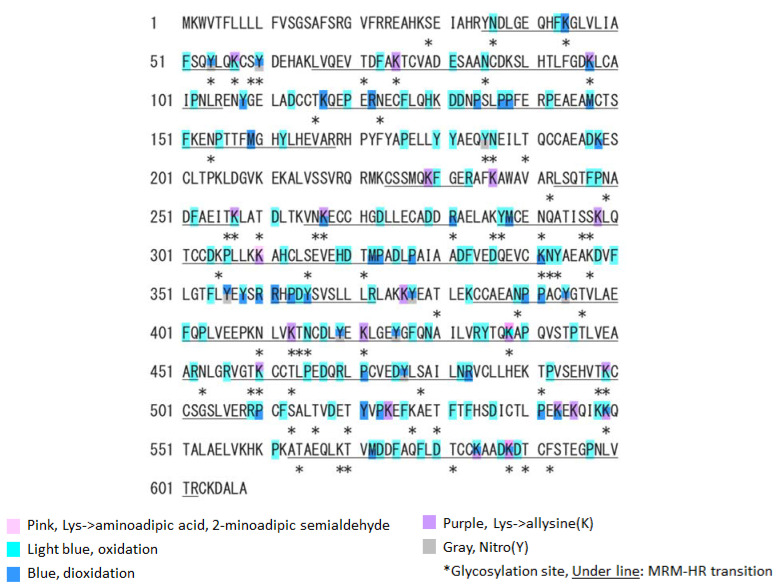
**Oxidative modification of SA obtained from single TBI exposure.** Identified MSA sequences and their oxidative modification by LS-MS/MS. The modification sites are marked as follows: pink, aminoadipic acid; light blue, oxidation; blue, dioxidation; yellow, γ-glutamyl semialdehyde; purple, allysine; and grey, nitrotyrosine. Glycated or glycosylated amino acids are indicated with asterisks. The peptides targeted by an MRM-HR are underlined.

**Figure 3:**
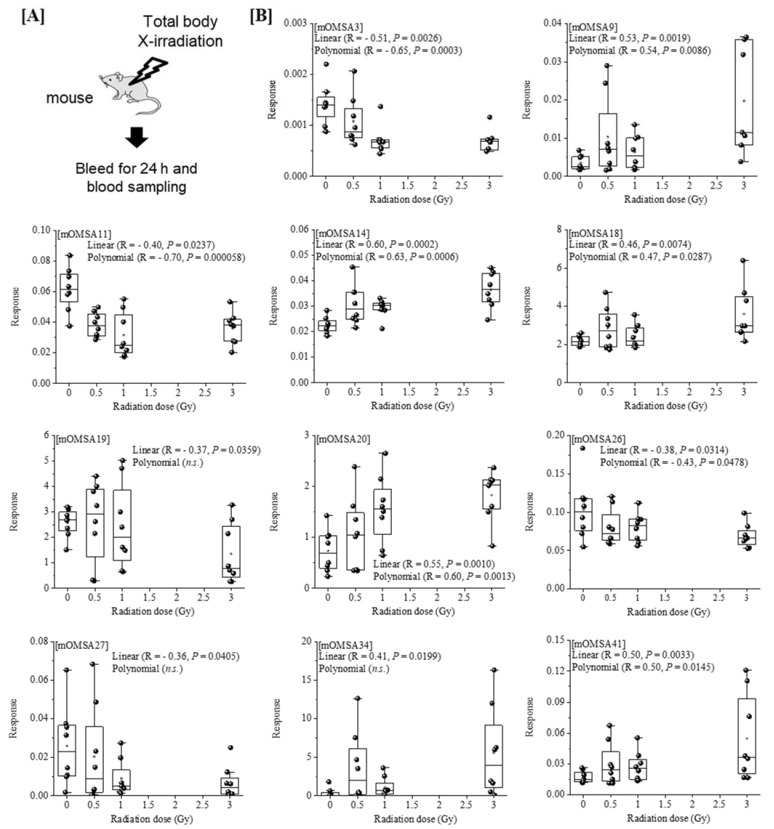
**Correlation between the oxidative modification sequence of MSA with single TBI and the radiation dose in vivo model.** [**A**] Eight-week-old female C57BL/6JJcl mice were exposed to varying TBI doses of 0, 0.5, 1 or 3 Gy of X-rays. Peripheral blood was harvested 24 h after TBI from the orbital venous plexus of mice (in vivo model). [**B**] Eleven sequences that showed significant dose-dependent correlations by linear or curve fitting are shown. Five sequences (mOMSA3, mOMSA9, mOMSA14, mOMSA20 and mOMSA41) showed a correlation coefficient (*r* value) of >0.5. *p* values of <0.05 were considered statistically significant.

**Figure 4:**
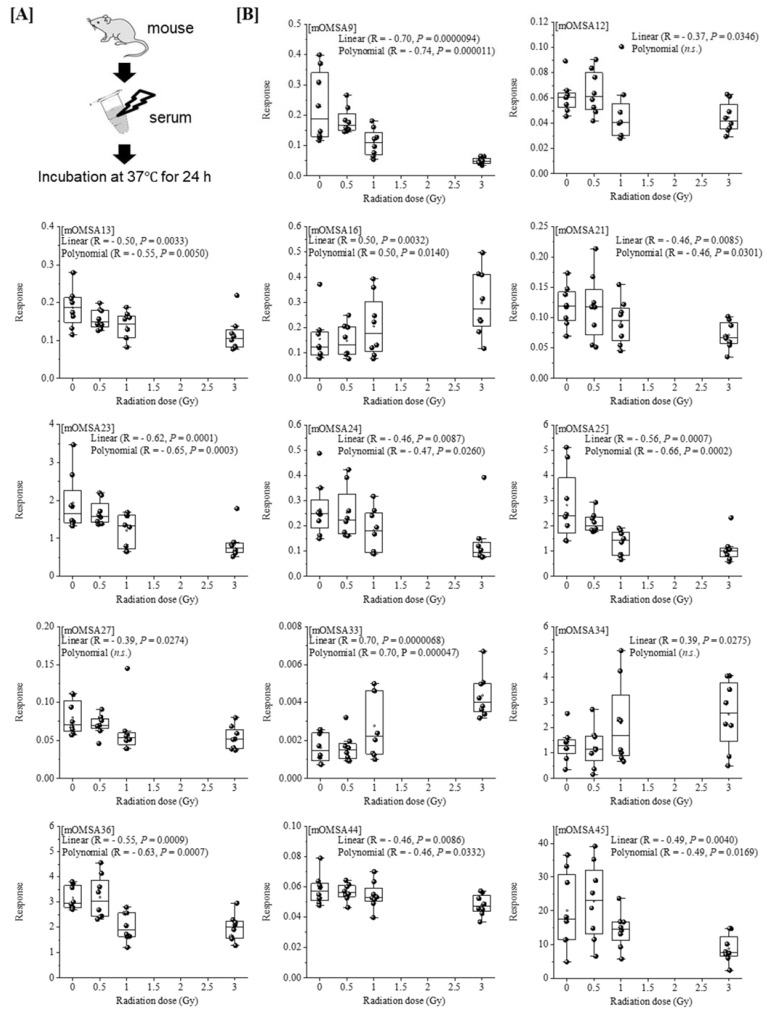
**Correlation between oxidative modification sequence of MSA irradiated in vitro.** [**A**] Serum samples collected from non-irradiated mice were subjecting to varying TBI doses of 0, 0.5, 1, or 3 Gy of X-rays and incubated at 37 °C for 24 h (in vitro model). [**B**] Fourteen sequences that showed significant dose-dependent correlations by linear or curve fitting are shown. Seven sequences (mOMSA9, mOMSA13, mOMSA16, mOMSA23, mOMSA25, mOMSA33 and mOMSA36) showed a correlation coefficient (*r* value) of >0.5. *p* values of <0.05 were considered statistically significant.

Next, we analyzed the oxidative modification of the chemical and spatial structure of albumin that occurred because of acute single radiation exposure. The amino acid sequence of mouse albumin and the identified modifications are shown in Figure 2. The albumin structural region was also totally glycated and oxidatively modified. In addition, nitration of the tyrosine residue and oxidation of the arginine residue, proline residue, methionine residue, and lysine residue were observed. The sequence information for 48 mouse OMSA (mOMSAs) is listed in Supplemental Table 1. For MRM-HR profiling of mOMSA, peptides containing each oxidatively modified amino acid residue were standardized against the peak area value of the corresponding unmodified peptide and analyzed for correlation with the radiation exposure dose as a relative peak area ratio. The fitting of quadratic equations was investigated for each mOMSA; in fact, most human genes show quadratic dose response to radiation [16,17]. In the in vivo model, eleven sequences showed significant dose-dependent correlations (*r* value) of >0.5 by linear or curve fitting. Especially, significant moderate or strong correlations were found between the individual acute high radiation exposure dose and five mOMSAs: mOMSA3 (Linear *r* = −0.51, *p* < 0.01, Polynomial *r* = −0.65, *p* < 0.001), mOMSA9 (Linear *r* = 0.53, *p* < 0.01, Polynomial *r* = 0.54, *p* < 0.01), mOMSA14 (Linear *r* = 0.6, *p* < 0.001, Polynomial *r* = 0.63, *p* < 0.001), mOMSA20 (Linear *r* = 0.55, *p* < 0.001, Polynomial *r* = 0.60, *p* < 0.01), and mOMSA41 (Linear *r* = 0.5, *p* < 0.01, Polynomial *r* = 0.5, *p* < 0.05) (Figure 3). Furthermore, serum samples collected from non-irradiated mice were subjected to varying TBI doses of 0, 0.5, 1, or 3 Gy of X-rays and incubated at 37 °C for 24 h as an in vitro model (Figure 4A). Fourteen sequences showed significant dose-dependent correlations (*r* value) of >0.5 by linear or curve fitting. Especially, significant moderate or strong correlations were found between the individual acute high radiation exposure dose and seven mOMSAs: mOMSA9 (Linear *r* = −0.7, *p* < 0.00001, Polynomial *r* = −0.74, *p* < 0.001), mOMSA13 (Linear *r* = −0.5, *p* < 0.01, Polynomial *r* = −0.55, *p* < 0.01), mOMSA16 (Linear *r* = 0.5, *p* < 0.01, Polynomial *r* = 0.5, *p* < 0.05), mOMSA23 (Linear *r* = −0.62, *p* < 0.001, Polynomial *r* = −0.65, *p* < 0.001), mOMSA25 (Linear *r* = −0.56, *p* < 0.001, Polynomial *r* = −0.66, *p* < 0.001), mOMSA33 (Linear *r* = 0.7, *p* < 0.00001, Polynomial *r* = 0.7, *p* < 0.0001), and mOMSA36 (Linear *r* = −0.55, *p* < 0.001, Polynomial *r* = −0.63, *p* < 0.001) (Figure 4B). 

**Figure 5:**
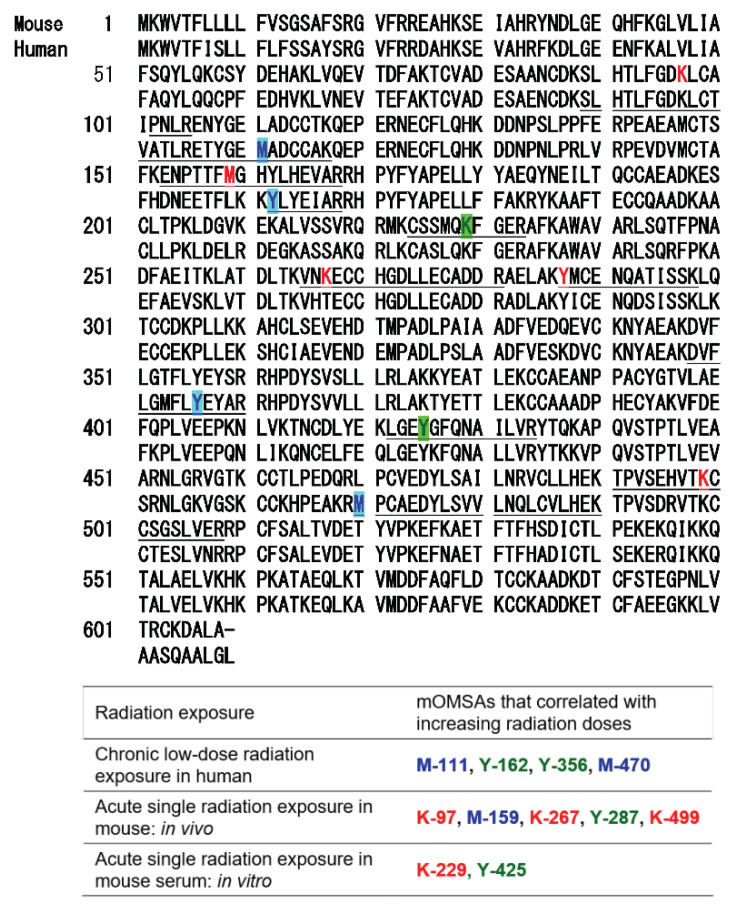
**Comparison of oxidative modification sequences of human and mouse serum albumin.** Based on the results of our previous report [1], the oxidative modification sites of the serum albumin obtained in this study were compared. Among the identified amino acid sequences of mouse albumin, lysine, methionine and tyrosine underwent dose-dependent oxidative modification. Oxidation-modified amino acids in chronic low-dose radiation exposure in humans, acute single radiation exposure in mice (in vivo), and acute single radiation exposure in mouse serum (in vitro).

Based on the results of our previous report [1], the oxidative modification sites of MSA obtained in this study were compared with the results of humans with chronic low-dose radiation exposure (Figure 5). Among the identified amino acid sequences of mouse albumin, lysine, methionine and tyrosine underwent dose-dependent oxidative modification. In particular, half of the oxidative modifications occurred at lysine, unlike in the case of human albumin (Table below in Figure 5). These results indicate that the profile of OMSA induced by radiation exposure is quite different between mice and humans.

## 4. Discussion

In the present study, a proteomic analysis of serum components from mice exposed to 0.5 to 3.0 Gy single TBI revealed significant, dose-dependent variation in six proteins (Table 1). Among these proteins, Serpin A8, Odorant-binding protein 1A, and PON1 were decreased, while other proteins were increased. In particular, the expression of Serpin A3K was found to increase in a dose-dependent manner (Supplemental Figure S1). Serpin A3K is a member of the serine protease inhibitor family and is also known as kallikrein-binding protein, with anti-inflammatory and anti-angiogenic activities [18]. This is a new finding, as no previous reports have shown an association between radiation and the expression of Serpin A3K. Similarly, Serpin A8, which is involved in blood pressure [19], and Odorant-binding protein 1A, which is involved in the sense of smell [20], have never been reported to be related to radiation, and this point was clarified for the first time in this study. Numerous reports on the association with radiation have been made for EGFR, which is a receptor of tyrosine kinase involved in cell survival/growth signaling that is overexpressed in several cancers [21,22]. In particular, EGFR is expressed in more than 90% of squamous cell carcinomas of the head and neck and is one of the most important therapeutic targets [23]. Following radiation, the activation of EGFR has been reported, leading to downstream signaling that contributes to cancer cell survival [24]. Further, EGFR has been shown to be involved in mediating DNA repair after irradiation, leading to the repair of damaged DNA [25]. There are also several reports on PON1 and prothrombin. Paraoxonase (PON-1) is an antioxidant enzyme that belongs to a family of calcium-dependent esterases that includes PON-1, PON-2 and PON-3 [26]. Serhatlioglu et al. examined the levels of malondialdehyde (an end-product of lipid peroxidation) and PON-1 activity/phenotypes in people, radiology workers, who were exposed to ionizing radiation for different time periods and doses [27]. They showed that PON-1 activity was reduced by 25–35% in subjects exposed to high-dose radiation (>3.5 mSv y^−1^) and in people with long−term exposure (>5 years) to radiation in comparison to the controls. Moustafa et al. evaluated the role of various enzymes in irradiated rats (6 Gy), demonstrating that the PON activity was significantly declined (*p* < 0.05) in comparison to the control group in both serum and the liver [26]. Similarly in this study, the PON1 value was reduced to 38% (Table 1). In addition, prothrombin, a glycoprotein (carbohydrate-protein compound) occurring in blood plasma and an essential component of the blood-clotting mechanism, is transformed into thrombin by a clotting factor known as factor X or prothrombinase [28]. Rithidech et al. reported an increase in prothrombin precursors in the plasma of irradiated (3 Gy) mice on day 2, suggesting an association with radiation [29]. As shown above, significant fluctuations in six serum proteins were observed 24 h after TBI (0.5–3.0 Gy) mice, suggesting that these molecules may be an effective biomarker in this exposure dose range.

**Figure 6:**
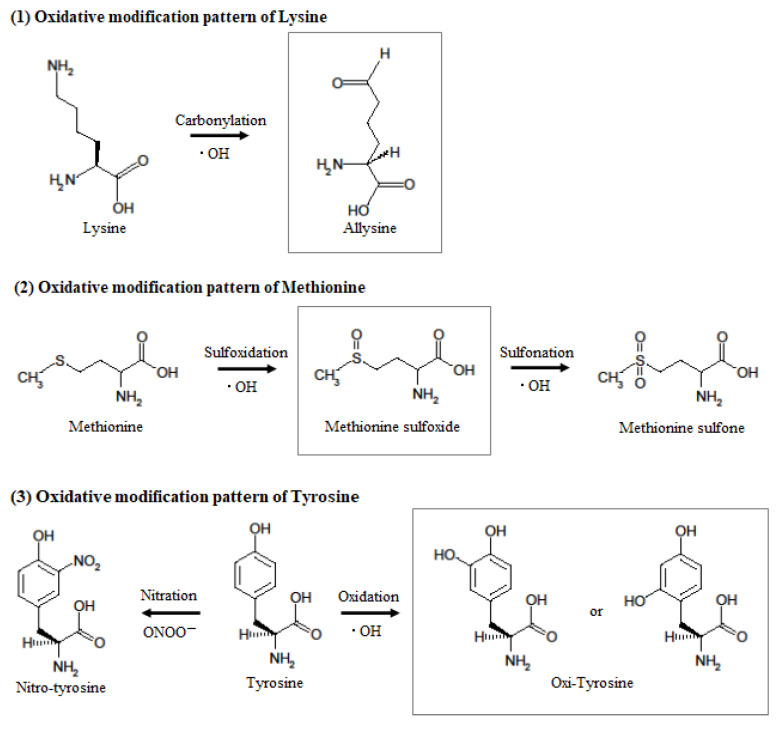
**Hypothesized mechanism of oxidative modification of mouse serum albumin after single TBI.** When tyrosine reacts with peroxynitrite or hydroxyl radicals, it becomes nitrotyrosine or hydroxytyrosine, but the aromatic substituent pattern cannot be identified by mass spectrometry. When methionine reacts with hydroxyl radicals, it becomes methionine sulfoxide and then methionine sulfone. When lysine reacts with hydroxyl radicals, it becomes allysine.

In our previous study, we developed an MRM-HR method targeting the 38 patterns of hOMSA using LS-MS/MS and performed the assay on serum samples collected from the residents of a newly discovered HBRA (annual effective dose approximately 50 mSv y^−1^) [1]. As a result, we reported a dose-dependent oxidative change in a specific sequence of human serum albumin. Dose-dependent oxidative modification of mouse serum albumin was observed in single total-body-irradiated mice as well as in the residents with chronic low-dose radiation exposure. In this study, four sequences (mOMSA9, mOMSA14, mOMSA20, and mOSMA41) in the in vivo model and two sequences (mOMSA16 and mOMSA33) in the in vitro model showed positive dose-dependent correlations, but one sequence (mOMSA3) in the in vivo model and five sequences (mOMSA9, mOMSA13, mOMSA23, mOMSA25, and mOMSA36) in the in vitro model showed negative dose-dependent correlations (r value) of >0.5 with *p* values of <0.05 by linear and curve fitting, suggesting that the correlation between the oxidative modification of MSA and the response to radiation differed between the in vivo and in vitro models (Figure 3 and Figure 4). It is well known that proteins circulating in the blood are one of the main targets of reactive oxygen species (ROS) produced by the interaction of ionizing radiation and water molecules. In addition, two prime suspects in the intracellular generation of ROS are also the membrane-bound NADPH oxidase complex and the mitochondrial electron-transport chain (ETC) in vivo model. Differences in the sources of ROS production in both models may contribute to the responsiveness to radiation exposure. Under the action of ROS, proteins undergo oxidative modification, leading to disruption of their structures and functions. Oxidatively damaged proteins accumulate during the course of ageing and under various pathological conditions [30]. In particular, serum albumin, which is present in mouse blood at a concentration of approximately 27 mg/mL, is the most abundant protein, accounting for approximately 54% of the plasma protein weight [31], and since it is exposed to various active chemical species at high frequency, it provides information on oxidative stress in systemic circulation. Of these amino acid residues, the amino acid lysine in proteins is subject to the largest variety of physiological post-translational modifications and is also among the most frequently carbonylated amino acids [32] (Figure 6). In this study as well, lysine carbonylation accounted for half of the identified serum albumin oxidation-modified sequence OMSA (Figure 5). Peroxynitrite (ONOO^−^) binds to the phenolic ring of tyrosine residues to produce nitrotyrosine. In addition, the methyl thioether group of methionine changes to a sulfoxide structure in response to increased levels of intracellular oxidative stress (Figure 6). Oxidative modification of serum albumin was also observed in mice after a single exposure to TBI, as it was in humans with chronic low-dose radiation exposure. Interestingly, mOMSA14, an oxidation-mediated modification site of the 159th methionine residue in the mouse albumin amino acid sequence, and an oxidation-mediated modification site of the 162nd tyrosine residue in the human albumin amino acid sequence, is located in domain IB of the albumin molecule (Figure 5). Although the profile of oxidative modification of albumin as a whole differs between humans and mice, it is noteworthy that the amino acid residues Met-159 in MSA and Tyr-162 in HSA, which correlate with exposure, are both located in domain IB of albumin [33]. The common oxidative modification response of domain IB of serum albumin to radiation suggests a possible link between the steric structure of the protein and the biological response to radiation and can be used as a biomarker of both acute and chronic radiation exposure for living organisms. Persistent non-physiological protein modifications, such as non-reparable oxidative protein carbonylation, are irreversible and mostly deleterious to protein activity, and expectedly to their interactions with partner molecules. However, the functional changes of oxidatively modified albumin, the subsequent effects on individual health conditions, diseases, and longevity, as well as the relationship with radiation damage, are issues to be addressed in the future.

Regarding the biological effects of ionizing radiation, initially, it was dominated by target theory, which quantifies the damage caused by traversal of cellular targets such as DNA by ionizing tracks [34,35]. Genomic DNA is the primary target, and double-strand breaks (DSBs) were found to be the most important radiation-induced DNA damage. DSBs differ from base excision repair in SSBs in that their repair pathway is more complex and requires more proteins. Later, the importance of “non-target” or “bystander” effects became recognized with the discovery that mutagenesis, death and/or altered behavior sometimes occur in cells that were not themselves traversed by any radiation tracks but which merely interacted with traversed cells [35,36]. A variety of short- and long-range cell-to-cell propagating signals have been reported, including small molecules capable of moving through gap junctions (e.g., lipid peroxide products, nucleotides), diffusible long-range signals such as proinflammatory cytokines (e.g., tumor necrosis factor-α) [37], and potentially micro RNAs [35] and exosomes [38]. Thus, various proteins are involved in radiation-induced damage and repair. On the other hand, the results of this study demonstrated the occurrence of dose-dependent protein oxidative modification by single TBI, revealing molecular damage to important proteins in targeting theory and non-targeted effects. Radman et al. reported that the first bottleneck in cell recovery from radiation damage is functional (proteome) rather than informational (DNA) [39,40]. They also indicated that although proteins and DNA are equally important for long-term survival, residual proteome activity after radiation stress can repair inactive damaged DNA and make it fit again for transcription and replication, but DNA cannot restore the proteome without pre-existing relevant protein activity. The results of this study–that single TBI causes many oxidative modifications to serum albumin in a dose-dependent manner–suggest that other proteins in the body undergo similar oxidative modifications. This means that, as Rithidech et al. postulated, changes in the expression levels of proteins may potentially be associated with late-occurring adverse effects [29]. Thus, protein variation and serum albumin oxidative modification responses found in exposed individuals are important indicators for considering the effects of radiation on living organisms along with DNA damage, in addition to their use as biomarkers estimation of the radiation dose.

## 5. Conclusions

Our previous report suggested that biological responses to chronic low-dose radiation in humans can be assessed by fluctuations in certain blood proteins and oxidative modification of HSA. The present results revealed significant increases or decreases in the serum levels of six proteins and demonstrated a dose-dependent oxidative modified region in serum albumin prepared from acute single TBI mice. Although the dose-dependent profiles of OMSA differed between acute single TBI in mice and chronic low-dose exposure in humans, the amino acid residues that correlate with exposure are all located in domain IB of albumin. It is interesting to note that the domains of albumin that are sensitive to oxidative reactions are consistent. These radiation responses are expected to have the potential to be used as biomarkers of acute and chronic radiation exposure in living organisms. DNA, the genetic material that holds all of the information of life phenomena, is an important biological target of ionizing radiation. Protein damage caused by ionizing radiation also needs to be considered in more detail.

## Figures and Tables

**Table 1:** Proteins that varied significantly according to the dose of X-irradiation.

Radiation Dose (Gy)	Protein Name	Protein Short Name	UniProt ID	FC ^1^	Probability ^2^
0 vs. 0.5	Angiotensinogen	Serpin A8	P11859	0.37	0.037
0 vs. 1.0	Odorant-binding protein 1a	Odorant- binding protein 1A	Q9D3H2	0.54	0.026
	Serine protease inhibitor A3K	Serpin A3K	P07759	1.84	0.026
0 vs. 3.0	Serum paraoxonase/ arylesterase 1	PON1	P52430	0.38	0.00076
	Prothrombin	-	P19221	2.04	0.0057
	Epidermal growth factor receptor	FGFR	Q01279	1.96	0.022
	Serine protease inhibitor A3K	Serpin A3K	P07759	2.54	0.046

^1^ FC, Fold change in comparison to non-irradiated samples. ^2^ Probability represents the *p* value determined by a *t*-test.

## Data Availability

All raw data for proteomics experiments are provided in supplementary information Table S1. The proteomics data are also available online using accession numbers “PXD025948, PXD025949, PXD025950” for Proteome Xchange [41] and accession numbers “JPST001166, JPST001167, JPST001168” for jPOST Repository [42]. Any additional data that support the findings of this study are available from the corresponding author upon reasonable request.

## Supplementary Materials

The following supporting information can be downloaded at: https://www.mdpi.com/article/10.3390/antiox11091710/s1, Figure S1: Correlation between the Serpin A3K expression levels with single TBI and the radiation dose.; Table S1: Oxidative modification pattern of peptide sequence consisting serum albumin in mice exposed to acute high dose/dose-rate radiation.
