# Supplementary material for: Serum Proteomic and Oxidative Modification Profiling in Mice Exposed to Total Body X-Irradiation
Source: Antioxidants (Basel). 2022 Aug 30;11(9):1710. doi: 10.3390/antiox11091710 (PMC9495380; doi:10.3390/antiox11091710)
Supplement: Supplementary file 1 [file antioxidants-11-01710-s001.zip › Supplemental Figure S1.pdf]

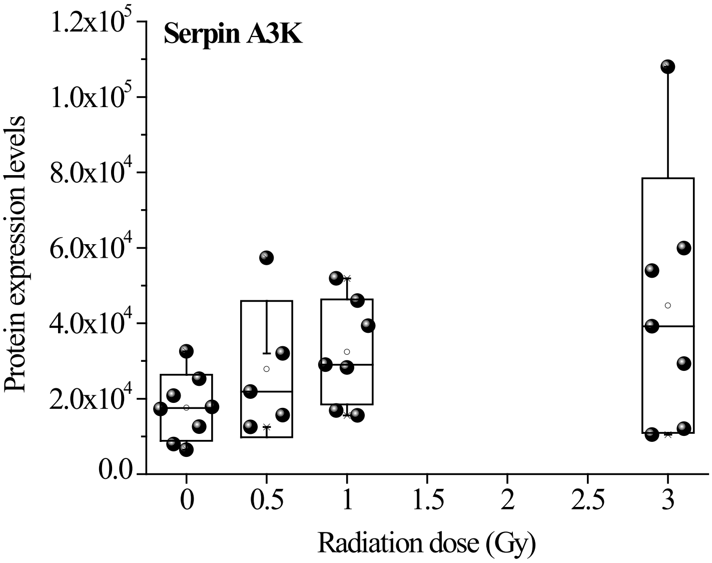

Supplemental Figure S1. Correlation between the Serpin A3K expression levels with single TBI and the radiation dose. A weak dose-dependent correlation was observed when the expression of serine protease inhibitor A3K (Serpin A3K) in non-TBI and TBI mice were compared. Linear (r=0.42, p=0.0176), polynormal (n.s.).
