# Supplementary material for: Serum Proteomic and Oxidative Modification Profiling in Mice Exposed to Total Body X-Irradiation
Source: Antioxidants (Basel). 2022 Aug 30;11(9):1710. doi: 10.3390/antiox11091710 (PMC9495380; doi:10.3390/antiox11091710)
Supplement: Supplementary file 1 [file antioxidants-11-01710-s001.zip › Supplemental Table S1.pdf]

Supplemental Table S1. Oxidative modification pattern of peptide sequence constituting serum albumin in mice exposed to acute high dose/dose-rate radiation

mOMSA ID	Peptide sequence and modification site	Residue number	Modification	Modification type
01	YNDLGEQHF[Oxi]K	43	F[Oxi]	Oxidation
02	YNDLGEQHFK[2Ox]	44	K[2Ox]	Oxidation
03	GLVLIAFSQY[Ntr]LQK	54	Y[Ntr]	Nitro-tyrosin
04	GLVLIAFSQY[Oxi]LQK	54	Y[Oxi]	Oxidation
05	GLVLIAFSQYLQK[AAA]	57	K[AAA]	Lys->Aminoadipicacid
06	GLVLIAFSQYLQK[LAA]	57	K[LAA]	Lys->Allysine
07	LVQEVTDFAK[LAA]TC[CAM]VADESAANC[CAM]DK	75	K[LAA]	Lys->Allysine
08	TC[CAM]VADESAANC[CAM]D[Oxi]K	87	D[Oxi]	Oxidation
09	SLHTLFGDK[LAA]LC[CAM]AIPNLR	97	K[LAA]	Lys->Allysine
10	SLHTLFGDK[2Ox]LC[CAM]AIPNLR	97	K[2Ox]	Oxidation
11	QEPER[2Ox]NEC[CAM]FLQHK	122	R[2Ox]	Oxidation
12	DDNPSLPPFERPEAEAM[Oxi]C[CAM]TSFK	147	M[Oxi]	Oxidation
13	DDNPSLPPFERPEAEAM[2Ox]C[CAM]TSFK	147	M[2Ox]	Oxidation
14	ENPTTFM[Oxi]GHYLHEVAR	159	M[Oxi]	Oxidation
15	ENPTTFM[2Ox]GHYLHEVAR	159	M[2Ox]	Oxidation
16	C[CAM]SSMQK[LAA]FGER	229	K[LAA]	Lys->Allysine
17	LSQTF[Oxi]PNADFAEITK	247	F[Oxi]	Oxidation
18	VNK[LAA]EC[CAM]C[CAM]HGDLLEC[CAM]ADDR	267	K[LAA]	Lys->Allysine
19	EC[CAM]C[CAM]HGDLLEC[CAM]AD[Oxi]DR	279	D[Oxi]	Oxidation
20	Y[2Ox]MC[CAM]ENQATISSK	287	Y[2Ox]	Oxidation
21	Y[Oxi]MC[CAM]ENQATISSK	287	Y[Oxi]	Oxidation
22	LQTC[CAM]C[CAM]DKP[Oxi]LLK	306	P[Oxi]	Oxidation
23	K[AAA]AHC[CAM]LSEVEHDTMPADLPAIAADFVEDQEVC[CAM]K	310	K[AAA]	Lys->Aminoadipicacid
24	K[LAA]AHC[CAM]LSEVEHDTMPADLPAIAADFVEDQEVC[CAM]K	310	K[LAA]	Lys->Allysine
25	K[Oxi]AHC[CAM]LSEVEHDTMPADLPAIAADFVEDQEVC[CAM]K	310	K[Oxi]	Oxidation
26	AHC[CAM]LSEVEHDTM[Oxi]PADLPAIAADFVEDQEVC[CAM]K	322	M[Oxi]	Oxidation
27	AHC[CAM]LSEVEHDTM[2Ox]PADLPAIAADFVEDQEVC[CAM]K	322	M[2Ox]	Oxidation
28	RHP[Oxi]DYSVSLLLR	363	P[Oxi]	Oxidation
29	C[CAM]C[CAM]AEANP[2Ox]PAC[CAM]YGTVLAEFQPLVEEPK	390	P[2Ox]	Oxidation
30	NLVK[LAA]TNC[CAM]DLYEK	413	K[LAA]	Lys->Allysine
31	LGEY[2Ox]GFQNAILVR	425	Y[2Ox]	Oxidation
32	LGE Y[Ntr]GFQNAILVR	425	Y[Ntr]	Nitro-tyrosin
33	LGEY[Oxi]GFQNAILVR	425	Y[Oxi]	Oxidation
34	YTQK[LAA]APQVSTPTLVEAAR	438	K[LAA]	Lys->Allysine
35	YTQKAP[Oxi]QVSTPTLVEAAR	440	P[Oxi]	Oxidation
36	VGTK[LAA]C[CAM]C[CAM]TLPEDQR	460	K[LAA]	Lys->Allysine
37	C[CAM]C[CAM]TLP[Oxi]EDQR	465	P[Oxi]	Oxidation
38	LPC[CAM]VEDY[2Ox]LSAILNR	476	Y[2Ox]	Oxidation
39	LPC[CAM]VEDY[Ntr]LSAILNR	476	Y[Ntr]	Nitro-tyrosin
40	LPC[CAM]VEDY[Oxi]LSAILNR	476	Y[Oxi]	Oxidation
41	TPVSEHVTK[LAA]C[CAM]C[CAM]SGSLVER	499	K[LAA]	Lys->Allysine
42	TPVSEHVTK[Oxi]C[CAM]C[CAM]SGSLVER	4999	K[Oxi]	Oxidation
43	ATAEQLKTVM[Oxi]DDFAQFLDTC[CAM]C[CAM]K	572	M[Oxi]	Oxidation
44	TVMDDF[Oxi]AQFLDTC[CAM]C[CAM]K	575	F[Oxi]	Oxidation
45	AADK[AAA]DTC[CAM]FSTEGPNLVTR	588	K[AAA]	Lys->Aminoadipicacid
46	AADK[LAA]DTC[CAM]FSTEGPNLVTR	588	K[LAA]	Lys->Allysine
47	AADKDTC[CAM]F[Oxi]STEGPNLVTR	592	F[Oxi]	Oxidation
48	AADKDTC[CAM]F[2Ox]STEGPNLVTR	592	F[2Ox]	Oxidation

Note . Starting on the left column, you can see “mouse OMSA (oxidative modification of serum albumin) ID”, “the Peptide sequence constituting serum albumin and its modification site”, and “the Modification type”, respectively.
